# Multielement Detection of Nonmetals by Barium-Based Post-ICP Chemical Ionization Coupled to Orbitrap-MS

**DOI:** 10.1021/jasms.3c00424

**Published:** 2024-04-23

**Authors:** Grace Hahm, Frenio A. Redeker, Kaveh Jorabchi

**Affiliations:** Department of Chemistry, Georgetown University, Washington, D.C. 20057, United States

## Abstract

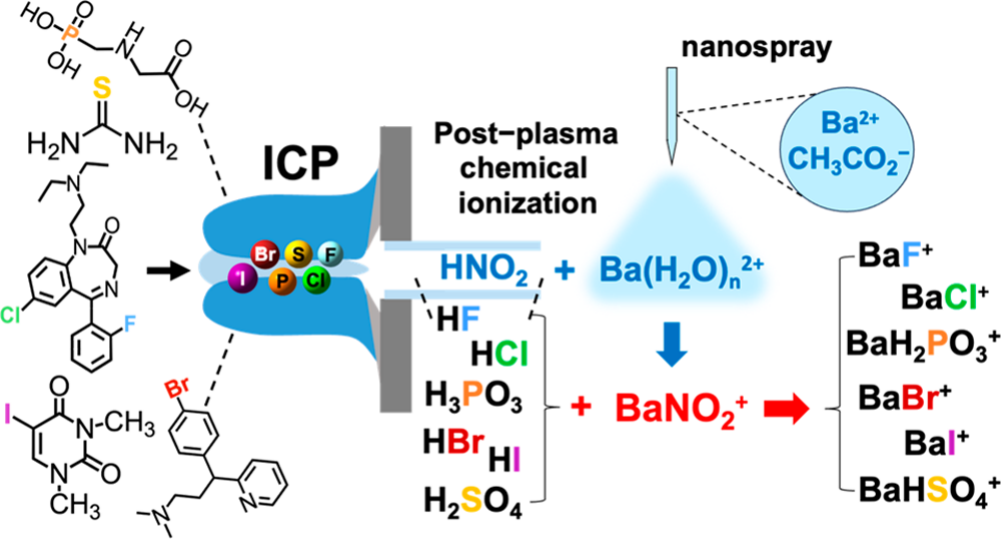

Prevalence of F, Cl, S, P, Br, and I in pharmaceuticals and environmental contaminants has promoted standard-free quantitation using analyte-independent heteroatom responses in inductively coupled plasma (ICP)-MS. However, in-plasma ionization challenges and element-dependent isobaric interference removal methods have hampered the multielement nonmetal detection in ICP-MS. Here, we examine an alternative approach to enhance multielement detection capabilities. Analytes are introduced into an ICP leading to post-plasma formation of HF, HCl, H_3_PO_3_, H_2_SO_4_, HBr, and HI, which are then chemically ionized to BaF^+^, BaCl^+^, BaH_2_PO_3_^+^, BaHSO_4_^+^, BaBr^+^, and BaI^+^ via reactions with barium-containing ions supplied by a nanospray. Subsequent ion detection by high-resolution MS provides an element-independent approach for resolving isobaric interferences. We show that elemental response factors using these ions are linear within 2 orders of magnitude and independent of analytes’ chemical structures. Using a single set of operating parameters, detection limits <1 ng/mL are obtained for Cl, Br, I, and P, while those for F and S are 1.8 and 6.2 ng/mL, respectively, offering improved multielement quantitation of nonmetals. Further, insights into ionization mechanisms indicate that the reactivities of reagent ions follow the order BaNO_2_^+^ > BaHCO_2_^+^ > Ba(H_2_O)_*n*_^2+^ ∼ BaCH_3_CO_2_^+^. Notably, the least reactive ions are generated directly by nanospray, suggesting that modification of these ions via interaction with plasma afterglow is critical for achieving good sensitivities. Moreover, our experiments indicate that the element-specific plasma products follow the order HF < H_2_SO_4_ ∼ HCl < H_3_PO_3_ ∼ HBr ∼ HI for their propensity to react with reagent ions. These insights provide guidelines to manage matrix effects and offer pathways to further improve the technique.

## Introduction

Molecular ionization methods, such as electrospray, are frequently used for detection and identification of xenobiotics and their transformation products. While immensely successful, analyte response factors in these methods are structure-dependent, requiring compound-specific standards for quantitation of the detected compounds. This creates roadblocks for absolute quantitation of compounds when species-specific standards are not readily available, e.g., in metabolite quantitation during new drug development. Radiolabeling the parent compounds is often used in such situations to quantitatively track the transformation products.^1^ However, this approach carries drawbacks of lengthy and expensive processes for synthesizing radiolabeled compounds, in addition to complications of handling radiolabeled materials. A recent approach has been introduced for standard-free absolute quantitation of molecules by combining coulometric and mass spectrometric measurements.^2^ However, this approach is limited to electroactive analytes (particularly in LC solvents when combined with chromatography) and requires characterization of all electrochemical products.

An attractive approach for standard-free quantitation is to utilize the heteroatoms in compounds as quantitative elemental tags. Notably, halogen (F, Cl, Br, and I), S, and P elements have been widely incorporated into drug motifs to enhance drug stability, metabolism, and selectivity.^3−6^ These heteroatoms are also prevalent in environmental contaminants (e.g., halogenated disinfection byproducts and phosphorylated flame retardants).^7,8^ Moreover, the appearance of F in drugs and environmental contaminants with health-related consequences has surged in the past decades.^9,10^ These considerations have fueled the development of elemental MS methods for F, Cl, Br, I, S, and P detection to offer species-independent calibration and rapid quantitation without compound-specific standards.^11−16^

Elemental MS methods most often utilize inductively coupled plasma (ICP)-MS, which relies on formation of atomic ions, i.e., S^+^, P^+^, Br^+^, I^+^, and Cl^+^, inside the high-temperature plasma. The formation efficiencies for these ions, therefore, follow the opposite order of their ionization potentials. These formation efficiencies are reflected in sensitivities (slopes of calibration curves). For example, ^35^Cl^+^ is detected with a sensitivity of 287 cps ng^–1^ mL (1.02 × 10^4^ cps μM^–1^ of Cl), about 1 order of magnitude lower than that for ^79^Br^+^ detected at 1550 cps ng^–1^ mL (1.24 × 10^5^ cps μM^–1^ of Br).^12^ As a consequence of the thermal ionization, the sensitivity for F^+^ detection is ∼10^4^-fold lower than that of Cl^+^,^17^ precluding use of this ion as a suitable analytical approach. Instead, recent efforts have tried to form BaF^+^ inside the ICP via simultaneous infusion of barium salts with analytes and operation of the plasma in cooler conditions to preserve BaF^+^ from dissociation.^15,18,19^ While improved compared to F^+^ detection, the F detection sensitivity via in-plasma BaF^+^ formation is ∼4 cps ng^–1^ mL (76 cps μM^–1^ of F),^19^ still 2 orders of magnitude lower than that of Cl^+^ detection. It is of note that formation of atomic ions requires a hot plasma, while BaF^+^ forms at cooler plasma temperatures. Thus, multielement methods with F measurement face a fundamental challenge in ICP-MS. Another complication for multielement methods relates to removal of isobaric interferences in ICP-MS. For most elements, ICP-MS/MS using O_2_ in the collision cell offers a suitable method to reduce isobaric interferences (e.g., via mass shift of S^+^ to SO^+^ to discriminate against O_2_^+^).^11^ In contrast, the best analytical performance for Cl^+^ detection is achieved via using H_2_ in the collision cell, yielding ClH_2_^+^.^12,13^

Overall, fundamental processes in ICP-MS reduce ion formation efficiency for F and Cl detection. This along with specificity of isobaric removal strategies via ion-neutral reactions make detection of F and Cl in a multielement method with other heteroatoms challenging. The multielement capability is notable because each heteroatom provides independent quantitation for compounds that have several measurable heteroatoms, adding an internal check for accuracy of quantitation results.^16^ Moreover, parts of analytes may be cleaved in transformation reactions, leading to loss of some heteroatoms. Therefore, a better coverage of transformation products is achieved with multielement methods.

To alleviate shortcomings of ICP-MS noted above, we have developed plasma-assisted reaction chemical ionization (PARCI) for elemental MS.^20−23^ In this approach, the analytes are introduced into a plasma, similar to ICP-MS; however, the ionization is not achieved inside the hot plasma. Instead, the plasma flow is allowed to cool significantly (<700 K),^24^ inducing recombination reactions and yielding element-specific polyatomic species. These species are then intersected with gaseous reagent ions for chemical ionization. We have recently reported that intersection of post-plasma flow with reagent ions generated from a nanospray of barium acetate electrolyte leads to formation of BaF^+^ and BaCl^+^ upon introduction of fluorinated and chlorinated compounds.^20^ The ion detection efficiency for F using this post-plasma BaF^+^ formation approach is 2 orders of magnitude higher than that of in-plasma formation used in ICP-MS.^21^ Moreover, post-plasma chemical ionization occurs at near room temperature compared to 1000s K in ICP-MS. This facilitates coupling pos-tplasma chemical ionization to various MS instruments. In particular, high-resolution (HR)-MS instruments provide a universal approach for elimination of isobaric interferences, further facilitating multielement detection methods as recently demonstrated for species-independent F and Cl quantitation by BaF^+^ and BaCl^+^ detection.^20^

In this work, we investigate the efficacy of Ba-based post-plasma chemical ionization with HR-MS detection as a general approach for multielement species-independent quantitation of S, P, Br, and I in addition to F and Cl. We characterize sensitivities, detection limits, and species-independent responses using this technique. Moreover, we provide insights into ionization pathways, further illuminating the potential of this approach for multielement detection of nonmetals.

## Experimental Section

### Chemical Ionization Interface

**Figure 1:**
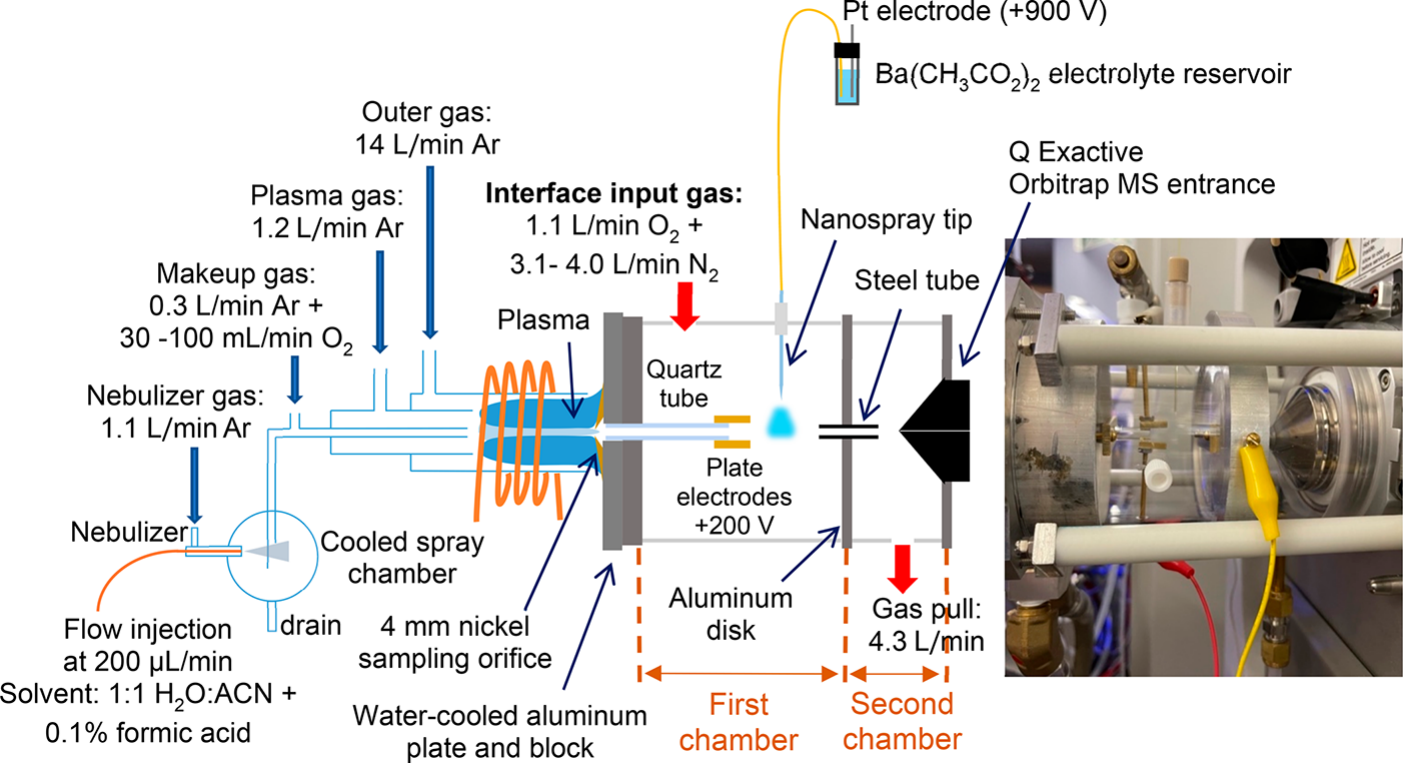
Schematic and a picture of a post-plasma chemical ionization interface fitted to a Q Exactive Orbitrap-MS. Note that Figure is not to scale.

An improved design of the post-plasma chemical ionization interface^25^ was utilized for better control of plasma sampling. This design was fitted to the entrance of a Q Exactive Orbitrap-MS as illustrated in Figure 1. The interface was composed of two chambers encased in acrylic cylinders (3 3/4″ o.d. × 3 3/4″ i.d.). The first acrylic cylinder (55 mm long) was pressed against a water-cooled aluminum block on the left side and a grounded aluminum disk on the right side. The second acrylic tube (50 mm long) was pressed against the aluminum disk on its left side, while its right side was pressed against the aluminum plate of the Q Exactive ion sampling interface. The assembly was held together using Delrin rods attached to the MS on one side and to the water-cooled aluminum block on the other side as depicted in the picture of Figure 1.

The cooled aluminum plate housed a 4 mm nickel sampler orifice (Spectron, Ventura, CA) in contact with the plasma. A quartz tube (1/4″ o.d., 4 mm i.d., 50 mm long) was placed ∼1 mm downstream of the plasma sampling orifice and was secured to the aluminum block using a graphite ferrule. The sampled plasma flow passing through the orifice traveled through the quartz tube, allowing cooling and induction of recombination reactions. A tightly fit steel tube (1/8″ o.d. × 2 mm i.d., 25 mm long) was incorporated into the grounded aluminum disk between the two chambers to enhance mixing of gases and ions prior to MS detection.

Plasma flow sampling was controlled by the gas evacuation from the second chamber and gas input into the first chamber. A constant flow of 4.3 L min^–1^ (monitored using a mass flow meter, TopTrak, Sierra Instruments, Monterey, CA) was evacuated from the bottom port of the second chamber. This evacuation rate, in addition to the MS flow sampling rate, induced a total of ∼5 L min^–1^ of gas flow from the first chamber into the second chamber through the steel tube. A varying flow rate of nitrogen mixed with 1.1 L min^–1^ oxygen was delivered into the first chamber using mass flow controllers (MKS Instruments Inc., Andover, MA). When the total interface input gas flow rate into the first chamber (summation of N_2_ and O_2_ gas flow rates) was larger than the gas flow rate evacuated through the steel tube, the excess gas flowed out of the plasma sampling orifice, precluding any plasma flow into the ionization interface. To increase the extent of plasma sampling, nitrogen gas flow rate into the first chamber was reduced. The total interface input gas flow rate was optimized by monitoring the analytical ion intensities as discussed in the Results and Discussion.

To ionize plasma-produced species, a borosilicate capillary (1 mm o.d., 0.75 mm i.d., World Precision Instruments, Sarasota, FL) was pulled to a tip size of ∼3 μm. The tip was then filled with 1 mM aqueous barium acetate solution. To sustain the spray for long operation, the electrolyte solution was also filled into a reservoir connected to the nanospray tip via a 100 μm i.d. fused silica capillary. The reservoir was placed at ∼15 cm above the tip, creating a gravity fed flow into the tip. The nanospray voltage (+900 V) was applied by inserting a platinum electrode into the reservoir solution. To guide ions toward the MS, parallel plate electrodes were installed at the end of the quartz tube with both plates held at +200 V. The addition of oxygen into the interface (mixed with nitrogen) was necessary for stability of the nanospray when plasma was sampled into the first chamber.

### ICP and Sample Introduction

The plasma was sustained in argon gas at radiofrequency power of 1300 W using a standalone generator (NexIon, PerkinElmer, Waltham, MA). The plasma torch was placed in front of the water-cooled plate such that the sampling orifice was 10 mm downstream of the load coil and concentric with the torch. The torch and the load coil were enclosed in a torch box, and a spring-loaded RF seal filled the spacing between the torch box and the aluminum water-cooled plate. The plasma gases were exhausted from the torch box into a hood using a 4″ inline fan (iPOWER, Duarte, CA).

All experiments were performed using 1:1 water:acetonitrile + 0.1% formic acid as solvent delivered at a flow rate of 200 μL min^–1^ using an HPLC pump to mimic typical reversed phase chromatographic conditions for 2.1 mm i.d. LC columns. Analytes were introduced via flow injections using a 50 μL PEEK loop. The sample flow was aerosolized by a nebulizer (HEN-90, Meinhard, Golden, CO) operated at 1.1 L min^–1^ of argon gas. Generated aerosols passed through a cyclonic spray chamber cooled to −2 °C by a Peltier cooler (PC^3X^, Elemental Scientific Inc., Omaha, NE). The emerging aerosol flow from the spray chamber was then mixed with a makeup gas composed of 0.3 L min^–1^ of argon and 30 to 100 mL min^–1^ of oxygen within a glass tangential mixer (Meinhard, Golden, CO). The resulting total aerosol flow was then guided into the ICP through the 2 mm i.d. injector of the ICP torch. The makeup oxygen flow rate was controlled using an electronic valve (Porter EPC, Parker Hannifin Corporation, Hartfield, PA) and an Arudino Due microcontroller board.

### MS Parameters

Ions were sampled into the Orbitrap-MS using a 150 °C ion transfer capillary and a setting of 50 for the S-lens. A 1 *m*/*z* SIM window was used for each analytical ion, and in-source CID was optimized for highest intensity. To expedite the data acquisition rate, the lowest resolving power allowing separation of isobaric interferences from each analytical ion was used in each SIM window. For this purpose, the SIM windows were first acquired at the maximum resolving power of 140 000 as depicted in Figure S1a–f to evaluate the extent of isobaric interferences. The resolving power was reduced to 17 500 for BaF^+^, BaCl^+^, BaBr^+^, and BaI^+^. Detection of BaH_2_PO_3_^+^, BaH_2_PO_4_^+^, and BaHSO_4_^+^ ions required the maximum resolving power. An AGC target of 1 × 10^5^ was used for all SIMs, except for P- and S-containing ions where AGC was reduced to 2 × 10^4^ to prevent the merging of interfering ions with analytical ions at high concentrations. To investigate background ions, MS scans were acquired in the range of *m*/*z* 50 to 500 with 35 000 resolving power setting and in-source CID of 20 eV.

### Chemicals and Sample Preparation

Neat standards were purchased from Sigma-Aldrich (Milwaukee, WI). Clindamycin phosphate, cyclophosphamide, iohexol, leflunomide, and levothyroxine were pharmaceutical secondary standards with certified purity ranging from 93.5 to 99.8%, whereas potassium iodide, chloramphenicol, glyphosate, thiourea, 5-bromouracil, and 5-iodo-1,3-dimethyluracil had a purity of ≥98%. Certified solution standards of 1 or 2 mg mL^–1^ concentration for cyanocobalamin (Vitamin B12), diltiazem HCl, bromazepam, brompheniramine, flunitrazepam, haloperidol, fluconazole, fluvoxamine maleate, flurazepam, chlorpromazine HCl, and fluphenazine 2HCl in methanol or acetonitrile were purchased from Cerilliant (Round Rock, TX). Bovine serum albumin protein (heat shock fraction, pH 7, with purity of ≥98%) was purchased from Sigma-Aldrich. 1-Palmitoyl-2-hydroxy-*sn*-glycero-3-phosphocholine (16:0 Lyso-PC) was from Avanti Polar Lipids. Acetonitrile, methanol, and formic acid were LC-MS grade and obtained from Thermofisher Scientific. *N*,*N*-Dimethylformamide (DMF) was purchased from Sigma-Aldrich. Ultrapure grade water (resistivity ≥18.2 MΩ.cm) was obtained from a Milli-Q Element water purification system.

For neat compounds, stock solutions were prepared by weighing >20 mg of neat standard and dissolving in about 10 to 13 mL of acetonitrile, water, or methanol. Nicergoline and levothyroxine standards were dissolved in DMF. Concentrations were then calculated by considering the purity. Working solutions were gravimetrically prepared by transferring aliquots of the stock solutions into 1:1 W:ACN solvent containing 0.1% formic acid. Nanospray solution of 1 mM barium acetate (ACS reagent, purity of 99%, Sigma-Aldrich, Milwaukee, WI) was prepared by dissolving neat compound in water.

### Data Analysis

Data were processed in R using the Tidyverse package.^26,27^ Orbitrap-MS data were converted into mzML format for processing in R with the MSnbase package.^28^ Visualization packages ggplot2 and cowplot in R were used to construct figure plots.^29,30^

## Results and Discussion

### Element-Specific Ions for Nonmetals

In our previous work, BaF^+^ and BaCl^+^ were identified as element-specific ions for F and Cl detection using barium-based chemical ionization in post-ICP.^20^ The formation of these ions was confirmed in this work using the enhanced chemical ionization interface described above (Figure 1) by flow injections of haloperidol. These ions were also used to optimize total interface input gas flow rate and plasma oxygen flow rate. We then introduced compounds containing P, S, Br, and I via flow injections to identify element-specific ions for these elements. Injection of glyphosate resulted in detection of BaH_2_PO_3_^+^ (*m*/*z* 218.8789, 0.23 ppm error) and BaH_2_PO_4_^+^ (*m*/*z* 234.8734, −1.58 ppm error), while injecting thiourea led to detection of BaHSO_4_^+^ (*m*/*z* 234.8642, −0.21 ppm error). Similarly, Ba^79^Br^+^ (*m*/*z* 216.8225, −2.5 ppm error) and Ba^81^Br^+^ (*m*/*z* 218.8209, −0.41 ppm error) were identified upon injections of 5-bromouracil, while BaI^+^ (*m*/*z* 264.8089, −1.0 ppm error) resulted from injection of 5-iodo-1,3-dimethyluracil.

These observations indicate that the post-plasma chemical ionization approach previously reported for F and Cl detection^20^ can be extended to other nonmetals. Insights into pathways for formation of these ions are presented in a later section of this report. Considering the goal of multielement nonmetal detection, we first investigated the effect of operating parameters on analytical ion intensities to evaluate the similarity of their optimal detection conditions. Further, we characterized the analytical performance at the optimum conditions.

### Similarity of Optimal Detection Conditions among Nonmetals

Ion intensities depend on two major factors in the post-ICP chemical ionization approach: (1) gas-phase concentration of plasma-produced species and (2) ionization efficiency of the species by the nanospray-produced reagent ions. Two experimental parameters in the setup of Figure 1 influence these factors: (1) total input gas flow rate supplied to the first chamber of the interface and (2) the oxygen flow rate introduced into the plasma. The effects of these parameters on ion intensities are discussed below.

**Figure 2:**
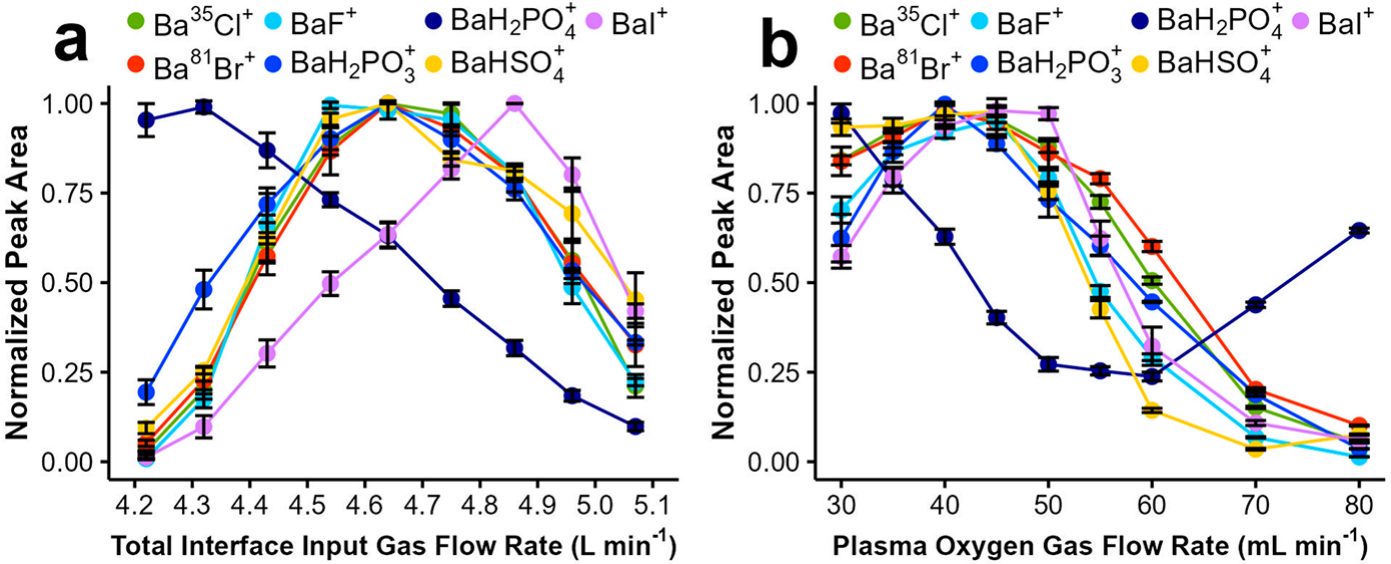
Effect of (a) total interface input gas flow rate and (b) oxygen gas flow rate introduced into the plasma on flow injection peak areas. Normalization to the highest value of peak area was implemented for each ion to facilitate comparison of the trends among ions. A constant plasma oxygen gas flow rate of 45 mL min^–1^ was used for panel (a), and a constant total interface gas flow rate of 4.7 L min^–1^ was used for panel (b). A solution mixture containing 9.9 μM elemental F (leflunomide), 4.1 μM elemental Cl (chloramphenicol), 1.0 μM elemental P (glyphosate), 48.3 μM elemental S (thiourea), 0.9 μM elemental Br (5-bromouracil) and 0.9 μM elemental I (5-iodo-1,3-dimethyluracil) was used. Error bars represent standard deviations based on triplicate flow injections.

Figure 2a shows the effect of total interface input gas flow rate (summation of N_2_ and O_2_ flow rates introduced in the first chamber in Figure 1) on flow injection peak areas for each analytical ion at a constant plasma oxygen flow rate of 45 mL min^–1^. The experiments started at the total interface flow rate of 5.05 L min^–1^. All ions show an initial increase in intensity as the interface gas flow rate is reduced. This is attributed to more efficient sampling of the plasma. The decrease in total input gas flow rate necessitates an increase in flow rate of the plasma gas through the orifice into the ionization chamber to balance the gas flow rate pulled through the steel tube. Accordingly, a more efficient transfer of the plasma products into the ionization area is expected at lower total interface input gas flow rates.

Notably, further reduction in interface input flow rate eventually leads to a reduction in intensity of ions in Figure 2a, suggesting existence of an opposing effect at lower flow rates supplied to the interface. As a result, generally a bell-shaped curve with an optimum flow rate is observed. We attribute this opposing effect to ionization efficiency, which will be further discussed in the ionization mechanism section. Importantly, most ions show a similar curve, indicating the potential for multielement analysis. Although the optimum flow rate for BaH_2_PO_4_^+^ ion detection occurs at lower values, formation of the BaH_2_PO_3_^+^ ion enables optimal P detection at the optimum operating setting for the other ions. On the other hand, optimal iodine detection using BaI^+^ requires a higher interface flow rate compared to that of other elements; therefore, a slightly compromised setting for this ion needs to be selected to allow for multielement detection of all ions at a single operating condition.

Figure 2b depicts the effect of the plasma oxygen flow rate on flow injection peak areas at a constant interface input gas flow rate. Introduction of oxygen into the plasma is necessary to prevent carbon deposition onto interface surfaces downstream of the plasma when organic solvents are used. However, as depicted in Figure 2b, this parameter also has a significant impact on ion detection, particularly at higher oxygen flow rates. Notably, all analytical ions, except BaH_2_PO_4_^+^, show a similar behavior with a nearly identical optimal oxygen flow rate. This further justifies use of BaH_2_PO_3_^+^ rather than BaH_2_PO_4_^+^ as the analytical ion for P detection in a multielement approach. The general loss of sensitivity at high oxygen flow rates is related to compromised ionization efficiency detailed later in this report. For the remainder of the studies, the plasma oxygen flow rate and interface input gas flow rate were optimized daily by monitoring all analytical ions using flow injections of a mixture of compounds. The optimal oxygen flow rate varied in the range of 40–45 mL min^–1^, while that for the interface flow rate was 4.3–4.7 L min^–1^.

In summary, the ion intensity behaviors as a function of the two critical operating parameters indicate that the post-plasma chemical ionization can be operated at a single set of operating parameters for detection of all six elements close to optimal conditions, significantly simplifying the operation for multielement quantitation of these nonmetals. Accordingly, we characterized analytical performance for detection of all six nonmetals at a single operating condition as the next step.

### Species-Independent Calibration and Analytical Performance

**Figure 3:**
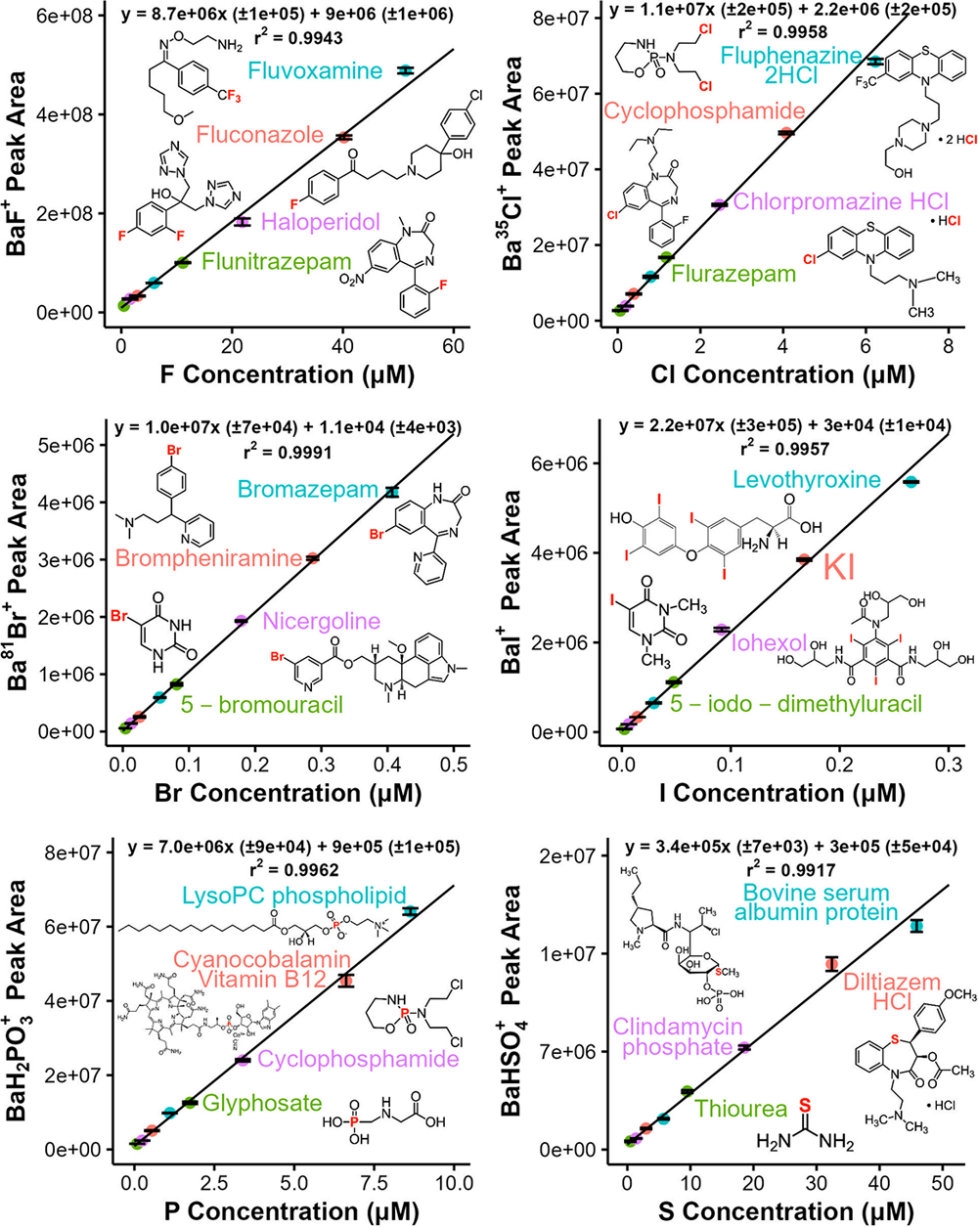
Element-specific calibration curves based on flow injection peak areas of compounds. Four compounds (indicated by data point color) were selected for each element, and each compound was injected at two concentrations. Average peak areas were weighted by 1/area for linear regression. Error bars represent standard deviations of peak areas of analytical ions based on triplicate flow injections. Total interface gas flow rate of 4.3 L min^–1^ and plasma oxygen gas flow rate of 45 mL min^–1^ were used in these experiments.

As noted in the Introduction, an appealing characteristic of elemental methods is the potential for species-independent response factors, enabling absolute quantitation of compounds without compound-specific standards. Ideally, a single calibration curve per element would be constructed based on this characteristic. To evaluate the potential of ICP-nanospray-HRMS in this regard, element-specific calibration curves were constructed using four compounds of varying structures per element as depicted in Figure 3. Each compound was injected at two different concentrations, leading to eight concentration levels per element. The concentration range for each element was selected to cover 2 orders of magnitude starting from the limit of quantitation. Figure 3 depicts weighted linear regressions with weights of 1/area spanning 2 orders of magnitude variation in elemental concentration. Notably, *r*^2^ values of 0.9917 to 0.9991 are obtained, indicating both good linearity and a compound-independent element-specific response. Thus, the data in Figure 3 demonstrate that ICP-nanospray-HRMS offers a single calibration curve per element for absolute quantitation of compounds regardless of analytes’ chemical structures.

It is of note that the slopes of calibration curves for halogens in Figure 3 vary only within a factor of 2. This is in stark contrast to orders of magnitude variation among halogen detection sensitives in ICP-MS, 0.49 cps μM^–1^ for F^+^, 76 cps μM^–1^ F using BaF^+^, 1.02 × 10^4^ cps μM^–1^ for Cl^+^, 1.24 × 10^5^ cps μM^–1^ for Br^+^, and 8.84 × 10^5^ cps μM^–1^ for I^+^.^12,17,19^ The similarities of sensitivities in ICP-nanospray-MS speak to the tunability of chemical ionization, overcoming the fundamental limitations of thermal ionization in ICP-MS. P detection also shows a similar sensitivity to those of halogens in Figure 3, while that of S is significantly lower compared to other elements. This is attributed to inefficiency of H_2_SO_4_ formation as detailed further in the ion formation mechanism discussions later in the report.

The experiments of Figure 3 were also designed to measure other analytical figures of merit, namely background equivalent concentrations (BECs) and limits of detection (LODs), for comparison to ICP-MS methods. The majority of ICP-MS methods utilize quadrupole-based instruments, and LODs are often measured using a 1 s integration time where each analytical ion impinges on the detector for a total of 1 s per data point. To produce similar detection conditions, we utilized an individual method for each calibration curve consisting of only one 1 *m*/*z* wide SIM window around the analytical ion. Further, the resolving power, automatic gain control level, and maximum ion injection times were adjusted to increase ion utilization efficiency and to improve precision (see details in Supporting Information). Finally, data points acquired within each 1 s were averaged in postprocessing, resulting in one data point per second for each analytical ion.

To calculate LOD and BEC, the baseline ion intensities and flow injection peak heights were utilized. Therefore, calibration curves of Figure 3 were reconstructed using flow injection peak heights to determine the slope. The BECs were then calculated via dividing the average baseline intensity (ion intensity prior to injection) by the slope of the flow injection peak height calibration curve, while the LODs were calculated using 3 × standard deviation of the baseline divided by this slope.

Table 1 summarizes the results for these metrics and compares them to representative values from ICP-MS/MS taken from references indicated in Table 1. The LOD for F detection with ICP-nanospray-HRMS is 10-fold better than that achieved by ICP-MS/MS, while the LODs for Cl, Br, and I are comparable to those of ICP-MS/MS methods. On the other hand, LODs for P and S are >10-fold higher than those using ICP-MS/MS, respectively. Considering that the high resolution of Orbitrap enabled separation of isobaric interferences, the BEC values for ICP-nanospray-HRMS reflect the levels of elemental contamination that originate from various sources such as solvents, tubing, and gases used in the experiment. These contaminations contribute to the elevated baseline and compromise LODs. For elements with significant BECs (F, Cl, S, and P), LODs are expected to be ∼10% of BEC assuming a precision of 3% RSD for baseline intensity measurement. Notably, the LODs for F and Cl are at 5 and 3% of their respective BECs, reflecting <10% RSD for baseline measurement precision. On the other hand, the LODs for P and S are at 20 and 70% of their respective BECs, reflecting compromised precisions. This is partly a result of ion utilization efficiency loss due to filling the C-trap with nonanalytical ions in the *m*/*z* windows for S and P detection, which is evident from lower actual ion injection times compared to maximum ion injection times in Table S1 for BaH_2_PO_3_^+^ and BaHSO_4_^+^. The situation is particularly exacerbated for S detection because of poor BaHSO_4_^+^ ion flux into the MS (evident from the lower calibration curve slope in Figure 3). Nevertheless, LODs are <1 ng mL^–1^ for majority of elements and in single digit ng mL^–1^ for F and S using a single set of plasma and ionization parameters, indicating the potential of this technique for high-sensitivity multielement detection.

**Table 1:** Instrumental Limits of Detection (LODs) and Background Equivalent Concentrations (BECs) Reported as ng mL^–1^ of Element

	ICP-nanospray-HR-MS			ICP-MS/MS				
Element	Ion	LOD	BEC	Transition	LOD	BEC	Solvent	Ref
F	BaF^+^	1.8	35	BaF^+^ BaF^+^	43	607^a^	H_2_O	(31)
Cl	Ba^35^Cl^+^	0.6	17	Cl^+^ ClH_2_^+^	1	52^b^	MeOH	(12)
Br	Ba^81^Br^+^	0.16	0.24	Br^+^ Br^+^	0.1	0.9^b^	MeOH	(16)
I	BaI^+^	0.08	0.057	I^+^ I^+^	0.03	0.4^b^	ACN	(16)
P	BaH_2_PO_3_^+^	0.4	2.1	P^+^ PO^+^	0.05	0.4	20% MeOH	(32)
S	BaHSO_4_^+^	6.2	8.9	S^+^ SO^+^	0.1	3.1	20% MeOH	(32)

a Calculated from ratio of baseline intensity to slope of calibration curve.

b Calculated from ratio of intercept to slope of calibration curve.

### Insights into Ion Formation Mechanisms via Competitive Ionization

The element-specific analytical ions are formed via reactions between barium-containing nanospray ions and plasma products. In other words, nanospray provides common reagent ions for chemical ionization. This commonality facilitates the method development for multielement analysis; however, it also implies potential for competition between ion formation pathways and consequently ion suppression at high concentrations of codetected nonmetals. To gain insights into ionization pathways and extent of ion suppression, we conducted competitive ionization experiments by monitoring the background ions and each analytical ion upon stepwise increases in concentration of other nonmetals to induce a suppression effect. The background ions were monitored to identify the key reagent ions for formation of each element-specific analytical ion.

**Figure 4:**
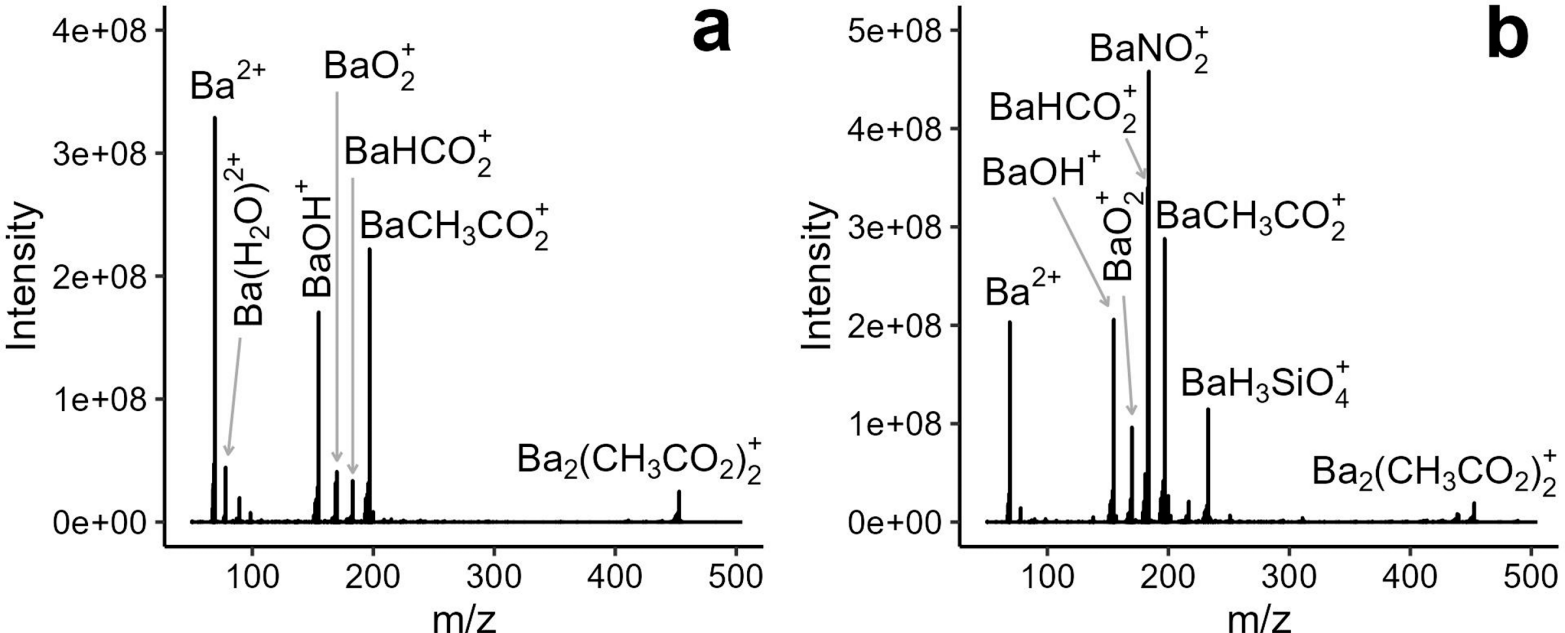
Background spectra from nanospray of a 1 mM aqueous barium acetate solution (a) without plasma sampling at a total interface input gas flow rate of 5.5 L min^–1^ and (b) with plasma sampling at a total interface input gas flow rate of 4.3 L min^–1^ and plasma oxygen flow rate of 45 mL min^–1^.

Figure 4a shows Ba^2+^ and BaCH_3_CO_2_^+^ as the main background ion species generated by nanospray when plasma is excluded from the ionization interface at high total interface gas flow rate. We note that an in-source CID of 20 eV is used to acquire the spectra in Figure 4 to decluster ions and reduce spectrum complexity. All ions are likely to exist primarily in their solvated forms in the ionization region. Ion activation by this in-source CID and by a capillary temperature of 150 °C and S-lens setting of 50 leads to BaOH^+^ and BaO_2_^+^ formation from fragmentation of solvated barium-containing ions. A small amount of BaHCO_2_^+^ is detected in the spectrum of Figure 4a, indicating residual contamination by formic acid. When the total interface input gas flow rate is reduced to that of the optimum operating conditions (Figure 4b), a significant reduction in Ba^2+^ is observed, while BaHCO_2_^+^ and BaNO_2_^+^ become the major ions in the spectrum, denoting conversion of original nanospray ions through reactions with plasma products. Excluding the fragmentation products (BaOH^+^ and BaO_2_^+^) in Figure 4b, we selected the major ions, namely Ba^2+^, BaCH_3_CO_2_^+^, BaNO_2_^+^, and BaHCO_2_^+^, as potential reagent ions for formation of element-specific analytical ions. We note that the BaH_3_SiO_4_^+^ ion is also detected in Figure 4b. We attribute this species to ionization of thermal degradation products of the quartz tube placed downstream of the plasma (Figure 1). The intensity of this ion was inconsistent in various experiments; therefore, we did not include this ion in our investigation of potential reagent ions.

Competitive ionization experiments were then conducted to characterize the ionization pathways. In these experiments, a matrix element was first selected, and a compound containing the matrix element was added with increasing concentrations to solutions containing an analyte element at constant concentration. These studies were performed in binary combinations, meaning only one matrix element and one analyte element were present in any given solution. The solutions were then introduced by flow injections while monitoring background ions and the ion corresponding to the analyte element in SIM mode. The ratio of the background ion intensity at the apex of the flow injection peak to that immediately prior to the injection was used to quantify the effect of matrix element on background ions. The analyte ion suppression in the presence of the matrix element was quantified by the ratio of the analyte ion response factor (flow injection peak area per element concentration) in the presence of the matrix element to that in the absence of the matrix.

**Figure 5:**
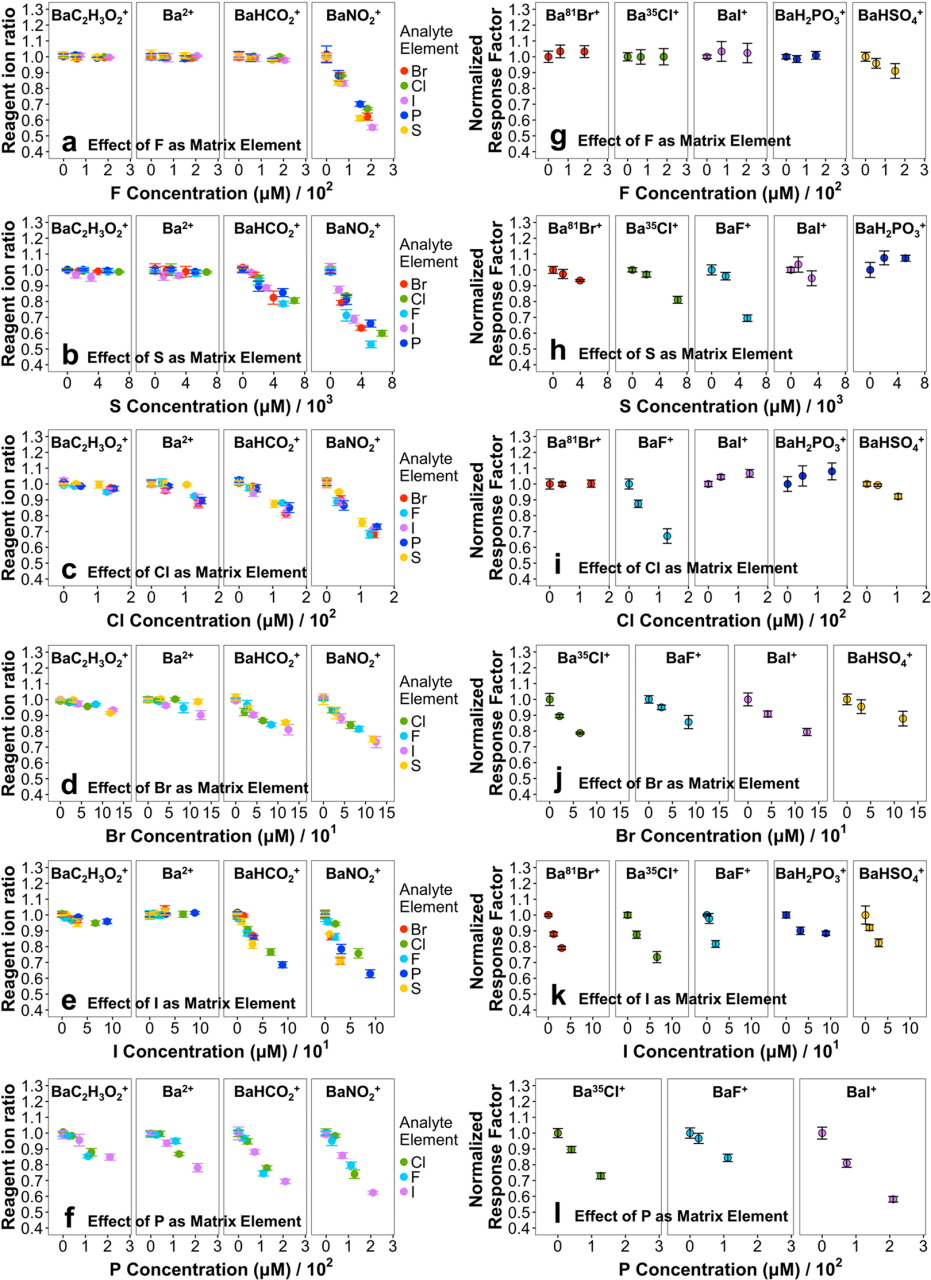
Effect of matrix elements on ion intensities of (a–f) major background ions and (g–l) other analytical ions. In panels (a–f), colors correspond to the analyte element used in the experiment. Error bars represent standard deviations of reagent ion ratios and response factors based on triplicate flow injections. A total interface input gas flow rate of 4.3 L min^–1^ and plasma oxygen gas flow rate of 45 mL min^–1^ were used for all measurements. Analytical ions were monitored using SIM windows with parameters in Table S1. Ba^2+^ and BaCH_3_CO_2_^+^ ions were measured with a 1 *m*/*z* wide SIM window, while a 3.0 *m*/*z* wide SIM window was used to monitor both BaHCO_2_^+^ and BaNO_2_^+^ with a resolving power of 17 500, AGC target of 1 × 10^5^, and maximum injection time of 80 ms. Matrix element concentrations were adjusted to observe a significant reduction in at least one of the background ions. Analyte element concentrations were selected based on the concentrations around the middle of respective calibration curves: 10 μM F, 3 μM Cl, 0.3 μM Br, 0.08 μM I, 5 μM P, and 20 μM S. Compounds for both analyte and matrix consisted of fluconazole or leflunomide for F, cyclophosphamide or chloramphenicol for Cl, 5-bromouracil for Br, 5-iodo-dimethyluracil for I, glyphosate for P, and thiourea for S.

We first consider the effects of matrix element concentration on background ions as depicted in Figure 5a–f in the order of increasing effectiveness of the matrix element to suppress background ions.

### Effect of F Matrix on Background Ions

Figure 5a shows that BaNO_2_^+^ is the only background ion undergoing significant reduction in intensity with increasing concentrations of F in sample. This selectivity to suppress BaNO_2_^+^ suggests that BaNO_2_^+^ is the key reagent ion for formation of BaF^+^. These observations also shed light on the nature of the plasma product reacting with BaNO_2_^+^ to yield BaF^+^. One can envision F^–^ and HF as potential plasma products of fluorinated compounds. Formation of BaF^+^ from F^–^ requires reaction with Ba^2+^ similar to that proposed for in-plasma formation of BaF^+^ in ICP-MS.^33^ However, ion-ion recombination of F^–^ with all positive ions is expected to be favorable and should also lead to neutralization of the singly charged ions in our experiments using ICP-nansopray-MS. Accordingly, one expects a reduction in all background ions and an even faster decline in Ba^2+^ due to faster reactions induced by higher charge, if F^–^ was the main plasma product of fluorinated compounds. In contrast, a strong selectivity for the BaNO_2_^+^ ion over other ions is observed in Figure 5a, suggesting that BaF^+^ results from ion-neutral reactions and that HF rather than F^–^ is the main plasma-product reacting with BaNO_2_^+^ as shown in Reaction 1:
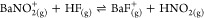
1It is of note that BaNO_2_^+^ itself is produced via interactions of the original nanospray ions, namely Ba^2+^ and BaCH_3_CO_2_^+^, with post-plasma flow (see Figure 4). Reactions 2 and 3 below show potential pathways for formation of BaNO_2_^+^ via reactions with plasma-produced HNO_2_.
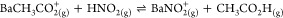
2
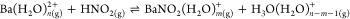
3Our previous computational results using density functional theory calculations at the theory level of ωB97xD/aug-ccpVTZ indicated an unfavorable thermochemistry for Reaction 2 (Δ*G*_298 K_ = 60.7 kJ mol^–1^), while a favorable thermochemistry was obtained for Reaction 3 with *n* = 3 and *m* = 2 (Δ*G*_298 K_ = −70.1 kJ mol^–1^).^21^ Another pathway for formation of BaNO_2_^+^ is the reaction of plasma-produced NO_2_^–^ with Ba(H_2_O)*_n_*^2+^. These considerations suggest that Ba(H_2_O)_*n*_^2+^ is the main precursor for BaNO_2_^+^. Interestingly, the direct reaction of Ba(H_2_O)_*n*_^2+^ with HF is not observed in Figure 5 in contrast to that of BaNO_2_^+^. Therefore, we conclude that reaction of Ba(H_2_O)_*n*_^2+^ with HF has a high activation barrier, likely resulting from charge separation along the reaction coordinate to produce two singly charged ions from a doubly charged ion, similar to that observed in fragmentations of hydrated Ba^2+^ ions.^34^ Consequently, formation of BaNO_2_^+^ facilitated by plasma flow interactions with nanospray ions can be considered as a catalytic step to convert the inactive Ba(H_2_O)_*n*_^2+^ to the reactive BaNO_2_^+^ reagent ion for HF ionization.

### Effects of Other Nonmetal Matrixes on Background Ions

The effect of the S matrix on background ions in Figure 5b also illustrates selectivity for singly charged ions, specifically BaNO_2_^+^ and BaHCO_2_^+^, thus denoting ion-neutral reactions of these ions with plasma-produced H_2_SO_4_ as the main ionization pathway for BaHSO_4_^+^ formation. Notably, the rate of ion intensity reduction for BaNO_2_^+^ is higher than that of BaHCO_2_^+^. This trend suggests that BaHCO_2_^+^ is the next most reactive reagent ion after BaNO_2_^+^ for ionization of H_2_SO_4_.

The effects of the Cl, Br, and I matrix on background ions are depicted in Figures 5c–e, further shedding light on the reactivity trends of background ions. In all cases, BaNO_2_^+^ and BaHCO_2_^+^ show faster reductions in intensity compared to those of BaCH_3_CO_2_^+^ and Ba^2+^. Similar to the case of F, these observations point to neutral species rather than ions as the plasma products of non-metal-containing compounds, indicating the main plasma products to be HCl, HBr, and HI for formation of BaCl^+^, BaBr^+^, and BaI^+^, respectively. Moreover, the rate of intensity reductions for background ions follows the order of BaNO_2_^+^ > BaHCO_2_^+^ > BaCH_3_CO_2_^+^ ∼ Ba^2+^ in Figures 5b–e, establishing relative effectiveness for these background ions to serve as reagent ions. Interestingly, the intensity reduction rates for BaNO_2_^+^ and BaHCO_2_^+^ become closer to each other in Figure 5d,e with Br and I matrix elements compared to the trends in Figure 5a–c with F, S, and Cl matrix elements. This observation suggests more efficient reactions of HBr and HI with BaNO_2_^+^ and BaHCO_2_^+^, perhaps approaching the collision rate.

The ion intensity reduction rates for BaCH_3_CO_2_^+^ and Ba^2+^ in Figure 5f for the P matrix also approach those of BaNO_2_^+^ and BaHCO_2_^+^, implying even more efficient reactions of reagent ions with the plasma products of P-containing analytes. Nevertheless, a preference for BaNO_2_^+^ and BaHCO_2_^+^ is still observed, which indicates that neutral species rather than ions constitute the main P-containing plasma products for formation of P-containing ions. Considering that both BaH_2_PO_4_^+^ and BaH_2_PO_3_^+^ are detected upon injection of P-containing compounds (see Figure 2), the intensity reductions in Figure 5f are attributed to a combined effect from reactions of background ions with both H_3_PO_4_ and H_3_PO_3_. In summary, investigation of background ion suppressions establishes HF, HCl, HI, HBr, H_2_SO_4_, H_3_PO_4_, and H_3_PO_3_ as the main element-specific species in the post-plasma flow for formation of analytical ions. Further, relative effectiveness of reagent ions is determined as BaNO_2_^+^ > BaHCO_2_^+^ > Ba^2+^ ∼ BaCH_3_CO_2_^+^.

### Relative Reactivity of Nonmetal Plasma Products and Analytical Ion Suppression

Relative reactivities of BaNO_2_^+^ and BaHCO_2_^+^ (the two most significantly affected ions) with each matrix element in Figure 5a–f also offer insights into relative reactivity order for element-specific neutrals. Reactions of HF with only BaNO_2_^+^ among ions denote HF as the least reactive species, while H_2_SO_4_ and HCl show reactions with both BaNO_2_^+^ and BaHCO_2_^+^. Finally, closer reactivities of BaNO_2_^+^ and BaHCO_2_^+^ with HI and HBr place these two plasma-products as the most reactive species. Thus, a reactivity order of HF < H_2_SO_4_ ∼ HCl < HBr ∼ HI for neutral plasma products can be deduced from Figure 5a–f. As noted above, the reduction in background ions by P-containing plasma products is a combined effect of H_3_PO_4_ and H_3_PO_3_, preventing placement of these species in the reactivity order. For hydrogen halides (HX), the order follows that of gas-phase acidity and shows the significance of deprotonation of acids in formation of corresponding BaX^+^. The lower reactivity of H_2_SO_4_ despite its high gas-phase acidity suggests involvement of other significant factors.

We now consider the effect of each matrix element on analytical ions in Figure 5g–l as complementary evidence for the mechanisms discussed above and to further shed light on suppression of analytical ions. Note that the data for effect of each matrix element on background ions in Figure 5a–f are collected simultaneously with those for the effects of matrix element on analytical ions in Figure 5g–l. Thus, the background ion and analytical ion Figures are shown side by side as pairs. Notably, the levels of suppression in background ions indicate the extent of ionization capacity overload by each matrix element.

Based on the reactivity order of nonmetal plasma products discussed above, we expect the F matrix that generates HF to have the least effect on other analytical ions. Indeed, Figure 5g confirms this prediction where increasing concentrations of F only affect BaHSO_4_^+^ and only slightly, while a major reduction in BaNO_2_^+^ is observed at the same conditions (Figure 5a). This observation suggests that the BaF^+^ replacing BaNO_2_^+^ serves as an effective reagent ion for ionization of H_2_SO_4_, HCl, HBr, HI, and H_3_PO_3_.

Figure 5h,i also denotes the similarity of matrix effects from S and Cl. Matrix concentrations that significantly reduce BaNO_2_^+^ and BaHCO_2_^+^ via reactions with H_2_SO_4_ and HCl result in the highest suppression of BaF^+^ among analytical ions followed by that of BaCl^+^ (S matrix in Figure 5h) and BaHSO_4_^+^ (Cl matrix in Figure 5i) while minimally affecting other ions. Interestingly, lack of BaH_2_PO_3_^+^ suppression by HCl and H_2_SO_4_ in Figure 5h,I allows placement of H_3_PO_3_ in the reactivity series as HF < H_2_SO_4_ ∼ HCl < H_3_PO_3_, augmenting the information derived from behaviors of background ions. Slight signal enhancements for BaH_2_PO_3_^+^ in Figure 5h,i and for BaI^+^ in Figure 5i are also noted, of which the origins are unclear at the moment and may relate to secondary factors such as transport efficiency of neutral plasma products through the quartz tube prior to reaching the ionization region (see Figure 1). Nevertheless, the relative slopes of analytical ion suppressions in Figure 5h,I follow the reactivity order discussed above.

Finally, Figure 5j–l illustrates the effects of Br, I, and P matrix elements on analytical ions. These elements lead to production of highly reactive HBr, HI, H_3_PO_3_, and H_3_PO_4_ as inferred from their effects on background ions. Thus, they are expected to suppress analytical ions of all other nonmetals. Importantly, Figure 5j–l confirms these expectations. Note that in the investigations of matrix effects, mutual effects of Br and P were not considered because of proximity of Ba^81^Br^+^ and BaH_2_PO_3_^+^ and proximity of BaPO_3_^+^ (likely fragmentation product of BaH_2_PO_4_^+^) and Ba^79^Br^+^, which resulted in overfilling of ion trap by the matrix ions, adversely affecting detection of the analytical ions. A similar ion trap overfilling effect by BaH_2_PO_4_^+^ prevented matrix effect studies of P on BaHSO_4_^+^.

Overall, the studies on background ions and analytical ions point to a similar order of reactivity for the nonmetal plasma products as HF < H_2_SO_4_ ∼ HCl < H_3_PO_4_ ∼ HBr ∼ HI and provide insights into detection sensitivity and potential of ion suppression in analytical measurements.

### Ionization Effects Induced by Interface Input Gas Flow Rate and Plasma Oxygen Flow Rate

**Figure 6:**
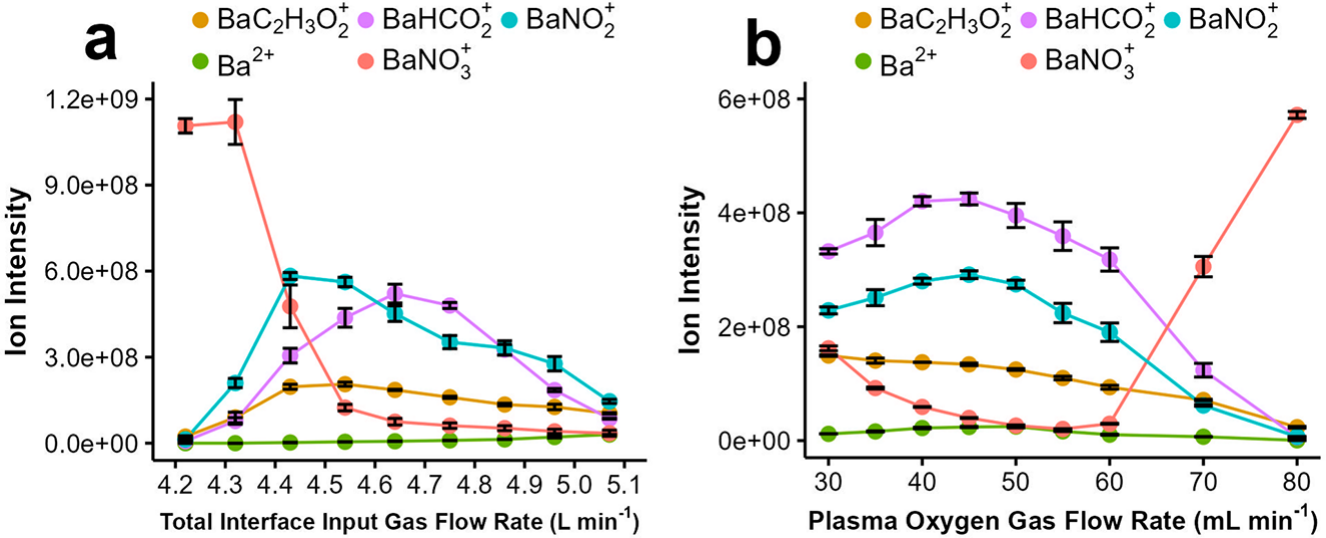
Effect of (a) total interface input gas flow rate and (b) plasma oxygen gas flow rate on intensities of background ions. Experimental conditions were identical to those described in Figure 2. Averaged intensities of background ions were extracted from a scan window of *m*/*z* 50 to 500 range. Error bars represent standard deviations of three measurements each collected for 30 s.

With the insights gained above regarding ionization pathways, we now return to the effects of plasma oxygen and interface input gas flow rates on ion intensities depicted in Figure 2. Figure 6 illustrates the effect of total interface input gas flow rate and plasma oxygen flow rate on the major background ions including an additional ion, BaNO_3_^+^. Notably, effective reagent ions identified above (BaNO_2_^+^ and BaHCO_2_^+^) show a bell-shaped behavior similar to those of analytical ions in Figure 2. This observation suggests that at least part of the bell-shaped behavior in Figure 2 is related to ionization effects. Interestingly, a rapid increase in BaNO_3_^+^ is observed at low interface gas flow rates and high oxygen gas flow rates, suppressing all other background ions in Figure 6. Similarly, suppression of the analytical ions is observed in these conditions in Figure 2. The increase in BaNO_3_^+^ ion formation stems from formation of HNO_3_ at low interface input gas flow rates and high plasma oxygen flow rates. The high reactivity of HNO_3_ leads to conversion of nearly all background ions to BaNO_3_^+^ as shown in Figure 6 (also see examples of mass spectra in Figure S2). As a result, analytical ions are also suppressed. In other words, operating conditions that produce abundant HNO_3_ leave poorly reactive BaNO_3_^+^ as the only reagent ion, resulting in loss of analytical sensitivity.

### Implications of Ionization Mechanisms in Analytical Performance

The reagent ion reactivity order of BaNO_2_^+^ > BaHCO_2_^+^ > Ba^2+^∼ BaCH_3_CO_2_^+^ along with detrimental effects of BaNO_3_^+^ provide insights into the impact of plasma chemistry on ionization efficiency. In other words, we posit that the plasma conditions that produce the highest intensities for BaNO_2_^+^ and BaHCO_2_^+^ would be most conducive to high-sensitivity detection of the six nonmetals. One factor affecting chemistry of the plasma is the balance of carbon and oxygen loadings, which varies in the course of gradient chromatography. Accordingly, a gradient of plasma oxygen flow rate synchronized with the solvent gradient must be used to optimize production of reagent ions.^20^

The reactivities of element-specific neutral plasma products are also important contributing factors to detection sensitivities of nonmetals. A higher reactivity is expected to increase detection efficiency and sensitivity. However, one must note that the detection efficiency is also affected by other factors including formation efficiency of nonmetal plasma products and biases for detection efficiency of analytical ions within the MS. The slopes of the calibration curves in Figure 3 follow the order BaHSO_4_^+^≪ BaH_3_PO_3_^+^ < BaF^+^ < BaCl^+^ < BaBr^+^ ∼ BaI^+^ when corrected for isotopic abundance of the ions. Interestingly, the sensitivity order for halogens follows that of the reactivity order established above as HF < HCl < HBr ∼ HI, indicating similar plasma product formation and MS detection efficiencies for these species. On the other hand, the sensitivity for BaH_2_PO_3_^+^ is lower than that expected from reactivity of H_3_PO_3_. However, we note that plasma products for P include H_3_PO_4_ in addition to H_3_PO_3_. Multiplicity of plasma reaction products reduces the concentration for each product, leading to reduced sensitivity. Strikingly, BaHSO_4_^+^ sensitivity is over 20-fold lower than that of BaF^+^, far lower than that expected from reactivity of H_2_SO_4_. We attribute this low sensitivity to lower efficiency of H_2_SO_4_ formation and transport to the ionization area. In other words, more efficient formation of other S-containing plasma products (e.g., SO_2_) not ionized by barium-based reagent ions could explain the low sensitivity for BaHSO_4_^+^.

In regard to ion suppression effects, high concentrations of P, Br, and I in samples are expected to cause the largest problems for detection of other nonmetals based on reactivities of the plasma products for these elements. Br and I are not prevalent biological elements; therefore, their presence in most samples is not expected to occur at high concentrations. However, P is prevalent, and its matrix effects should be considered carefully in analyses. Naturally, separations prior to elemental detection are used to quantify compounds; thus, better separations in conjunction with sample preparation methods could be utilized to minimize coelution of analytes of interest with high concentrations of P-containing compounds. Notably, the suitability of BaNO_2_^+^ as a reagent ion for all nonmetals offers a real time quality control metric to monitor the ionization. Any significant depletion of this ion during analyses may be used to flag the analyst for consideration of the matrix effects.

## Conclusions

The studies above provide an evaluation of post-plasma chemical ionization for multielement nonmetal detection from both analytical and fundamental perspectives. Analytically, we show that the technique offers multielement detection of Cl, Br, I, and P with LODs < 1 ng mL^–1^, while F and S are detected with LODs of 1.8 and 6.2 ng mL^–1^, respectively, in a solvent commonly used for reversed phase chromatography. Moreover, compound-independent elemental responses are obtained, which are critical for quantitation of compounds without standards. The 10-fold better LOD for F compared to that of ICP-MS/MS is particularly notable considering the rising significance of this element in biological and environmental analyses.

In terms of fundamental insights, we show that the enhanced analytical performance for multielement detection is enabled by formation of effective reagent ions, namely BaNO_2_^+^ and BaHCO_2_^+^, to ionize acidic plasma products of nonmetals. Notably, these effective reagent ions are produced by reactions of plasma products with original nanospray ions, largely Ba(H_2_O)_*n*_^2+^, while these original ions show low reactivity toward most element-specific plasma products. Therefore, tuning the plasma chemistry (e.g., via balancing the carbon to oxygen ratio) and control on plasma sampling extent play critical roles to ensure efficient conversion of original nanospray ions to more reactive species for enhanced ionization efficiencies.

These fundamental insights provide practical approaches for improved analytical performance and offer areas to focus for further technique improvements. For example, an oxygen gradient with reversed phased chromatography can help tune plasma chemistry and maintain the optimal reagent ion production. Further, real time monitoring of BaNO_2_^+^ can be used for quality control to flag matrix effects without a priori knowledge of the sample matrix. Ideally, new ionization chemistries should be developed to address these areas in a fundamental fashion. For example, we have recently demonstrated that Sc(NO_3_)_2_(H_2_O)*_n_*^+^ acts as a more selective reagent ion for HF detection.^25^ As a result, the HF detection dependence on plasma chemistry is reduced, and tolerance for matrix effects from Cl is improved. Similar selective chemistries for detection of other nonmetals are needed and are under development in our group to enhance multielement methods for robust and facile quantitation of compounds without standards.

## Supporting Information Available

High-resolution mass spectra for the 1 *m*/*z* SIM window around analytical ions at baseline level, MS parameters for calibration curves and analytical figures of merit, background spectra for operating conditions of low interface total input gas flow and high plasma oxygen gas flow (PDF)
