# Supplementary material for: Multielement Detection of Nonmetals by Barium-Based Post-ICP Chemical Ionization Coupled to Orbitrap-MS
Source: J Am Soc Mass Spectrom. 2024 Apr 23;35(5):871–82. doi: 10.1021/jasms.3c00424 (PMC11066957; doi:10.1021/jasms.3c00424)
Supplement: Supplementary file 1 — js3c00424_si_001.pdf [file js3c00424_si_001.pdf]

Supporting Information

Multielement Detection of Nonmetals by Barium-Based Post-ICP Chemical Ionization Coupled to Orbitrap-MS

Grace Hahm, Frenio A. Redeker, and Kaveh Jorabchi*

Department of Chemistry, Georgetown University, Washington, DC 20057

*Corresponding author: kj256@georgetown.edu

Table of Contents

High-resolution mass spectra for 1-m/z windows around analytical ions (Figure S1)	Page S2
Experimental parameters for analytical figures of merit	Page S3
Table S1. Summary of MS parameters for determining analytical figures of merit	Page S4
Background mass spectra at low total interface input gas flow rate and high plasma oxygen flow rate (Figure S2)	Page S4

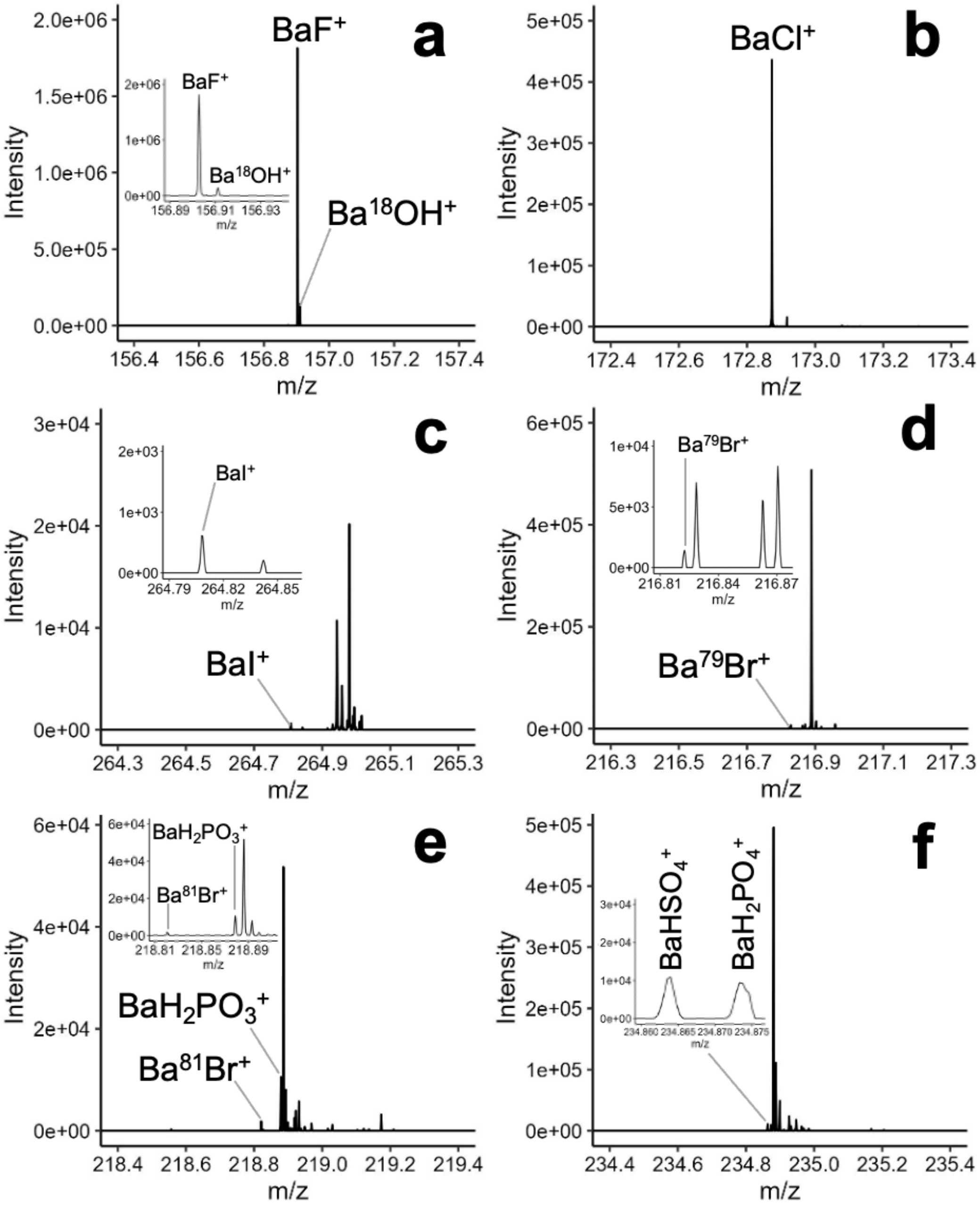

Figure S1. Mass spectra of 1.0-*m/z* windows for detection of **a)** $^{138}BaF^+$ (*m/z* 156.9031, 0 ppm error), **b)** $^{138}Ba^{35}Cl^+$ (*m/z* 172.8734, −0.87 ppm error), **c)** $^{138}BaI^+$ (*m/z* 264.8089, −1.0 ppm error), **d)** $^{138}Ba^{79}Br^+$ (*m/z* 216.8225, −2.5 ppm error), **e)** $^{138}Ba^{81}Br^+$ (*m/z* 218.8209, −0.41 ppm error) and $^{138}BaH_2PO_3^+$ (*m/z* 218.8789, 0.23 ppm), **f)** $^{138}BaH_2PO_4^+$ (*m/z* 234.8734, −1.58 ppm error) and $^{138}BaH^{32}SO_4^+$ (*m/z* 234.8642, −0.21 ppm error). Mass spectra were collected at baseline level without any injection of analytes to identify interfering isobaric ions. Highest resolving power setting of 140000 with maximum injection time of 500 ms and AGC target level of 2×10^4 were applied in the acquisition of the spectra. In source CID of 50 eV were used in measurements for $^{138}BaF^+$ and $^{138}Ba^{35}Cl^+$, and an in-source CID of 20 eV was used for all other ions. Constant total interface gas flow rate of 4.7 L min^{-1} and plasma oxygen gas flow rate of 45 mL min^{-1} were used.

MS parameters for calibration curves and for determining analytical figures of merit

The experimental parameters for detection of ions are listed in Table S1. First, the lowest resolving power allowing the separation of analytical ions from nearby interfering ions was selected. The lower resolving powers allow faster operation of the Orbitrap (see readout frequency in Table S1), therefore, more scans can be collected and averaged during the 1 second total measurement time per data point. To determine the lowest resolving power, each analytical ion was investigated for isobaric interferences, as shown in Figures S1a-f. These experiments were performed using the maximum resolving power of 140000, with lowest AGC target levels of 2×10^4 to prevent any merging of ions by overfilling the trap. For BaF^+ in Figure S1a, $Ba^{18}OH^+$ is the only major interference in the spectrum. For $BaCl^+$ in Figure S1b**,** a small neighboring peak at *m/z* 172.8700 is observed, however, this peak is insignificant compared to the background level of $BaCl^+$. As a result of minimal interfering ions and the significance of BaF^+ and $BaCl^+$ baseline levels relative to any other ions detected in the mass spectra windows, the lowest resolving power setting of 17500 was selected for these ions. For BaI^+, a setting of 17500 was sufficient to resolve the isobaric interferences as shown in Figure S1c. For the mass spectrum of $Ba^{79}Br^+$ in Figure S1d, a large neighboring peak at *m/z* 216.8285 close to $Ba^{79}Br^+$ was detected, whereas a major interfering peak was not detected for $Ba^{81}Br^+$ in Figure S1e. Therefore, $Ba^{81}Br^+$ ion was selected to monitor as the analytical ion for Br with a resolving power setting of 17500. Also shown in Figure S1e**,** is $BaH_2PO_3^+$ ion, which requires high resolving power of 140000 for separation from a dominant background ion at *m/z* 218.8861. Similarly, for $BaHSO_4^+$ analytical ion in Figure S1f, the highest resolving power setting of 140000 was needed to separate isobaric interferences.

To increase ion utilization efficiency and precision, the maximum ion injection times were set to the Orbitrap readout times. In ideal conditions, this setting would allow storage of the full ion flux in the C-trap while Orbitrap analyzes the previous batch of the ions. However, the actual ion injection times are also determined by the automatic gain control (AGC) level. An AGC of 1×10^5 is typically recommended for 1-m/z windows to avoid ion trap overfilling which would cause mass shift and resolving power deterioration. For BaF^+, $BaCl^+$, $BaBr^+$ and BaI^+ ions, this AGC level was adequate for separation of analytical ions from interferences. Table S1 shows that this setting resulted in actual ion injection times similar to the maximum injection time setting. Accordingly, for these ions, the ion utilization efficiency at the baseline (without any analyte injection) approached 100% in a method with only one SIM window, similar to that of quadrupole instruments used in ICP-MS. On the other hand, for $BaH_2PO_3^+$ and $BaHSO_4^+$ ions, AGC target values of $> 2 \times 10^4$ resulted in the analytical ions merging with the adjacent peaks upon injections of analytes, limiting the AGC target value for these ions. This constraint reduced the ion utilization efficiency for these ions evident from the lower actual ion injection times compared to maximum injection time setting in Table S1.

**Element-Specific Ion**	**CID (eV)**	**Resolving Power Setting**	**Orbitrap readout frequency (Hz)**	**Maximum Ion Injection Time (ms)**	**Typical Ion Injection Time (ms)***	**AGC target**
$^{138}BaF^+$	50	17500	12	80	80	1×10^5
$^{138}Ba^{35}Cl^+$	50	17500	12	80	80	1×10^5
$^{138}Ba^{81}Br^+$	20	17500	12	80	80	1×10^5
$^{138}BaI^+$	20	17500	12	80	80	1×10^5
$^{138}BaH_2PO_3^+$	20	140000	2	500	203	2×10^4
$^{138}BaH^{32}SO_4^+$	20	140000	2	500	102	2×10^4

Table S1. Mass spectrometer operating parameters for calibration curve and figures of merit

* values reflect measurements at baseline intensities without analyte injections

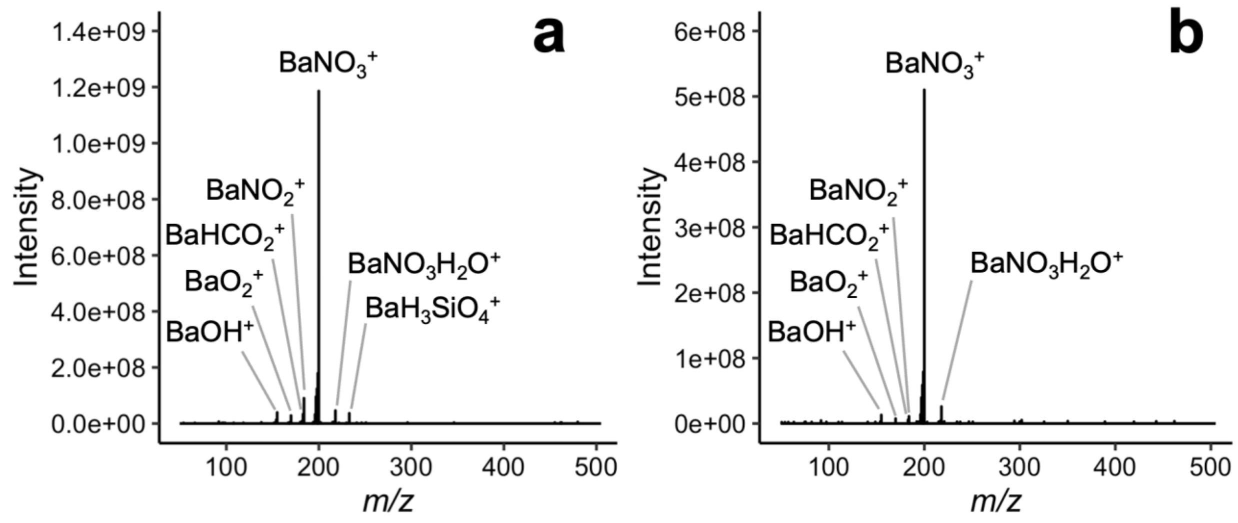

Figure S2. Background mass spectra corresponding to Figures 2 and 6 at **a)** low total interface gas flow rate of 4.3 L min^{-1} and optimal oxygen flow rate of 45 mL min^{-1} **b)** optimal interface flow rate of 4.6 L min^{-1} and high plasma oxygen flow rate of 80 mL min^{-1}.
